# Assessment of pulpal changes in periodontitis patients using CBCT: a volumetric analysis

**DOI:** 10.3389/fdmed.2025.1549281

**Published:** 2025-06-19

**Authors:** Ammar Almarghlani, Reem A. Alsahafi, Fatimah K. Alqahtani, Yousef Alnowailaty, Mohammed Barayan, Ameerah Aladwani, Amr Bokhari

**Affiliations:** ^1^ Faculty of Dentistry, King Abdulaziz University, Jeddah, Saudi Arabia; ^2^ General Dentist, King Abdulaziz University, Jeddah, Saudi Arabia; ^3^ Oral and Maxillofacial Radiology, Oral Diagnostic Science Department, King Abdulaziz University, Jeddah, Saudi Arabia; ^4^ My Clinic, Dental Center, Jeddah, Saudi Arabia; ^5^ Periodontics Department, King Abdulaziz University, Jeddah, Saudi Arabia

**Keywords:** endodontitis, periodontal lesions, cone-beam computed tomography, CBCT, periodontal teeth

## Abstract

**Introduction:**

Evidence suggests that periodontal disease can lead to inflammation and degeneration within dental pulp, highlighting the need for dental professionals to understand this association better.

**Objective:**

The objective for this study was to establish a correlation between pulp volume and periodontal disease using Cone-Beam Computed Tomography (CBCT) imaging.

**Methods:**

A cross-sectional study design was employed for the collected data from 148 patients aged 39.51 years using dental images obtained by CBCT and analyzed by medical software to create three-dimensional (3D) images. Pulp-volume analysis was performed using Amira software and measurements were derived using bio-models generated from CBCT images. Obtained data was analyzed using SPSS-27 statistical software.

**Results:**

The mean pulp volume between healthy and teeth with periodontitis showed certain differences. The mean low and largest pulp volumes of 9.15 ± 3.3 mm^3^ and 15.24 ± 4.2 mm^3^ were observed involving teeth # 41 and teeth # 13, respectively. Furthermore, a higher mean of pulp volume was observed in healthy teeth than in periodontitis-diagnosed teeth except for teeth # 33 and 43. The significant difference (*p* < 0.05) was easily detected involving teeth # 22, 23, 11, and 13. However, the lowest difference, with non-significant difference (*p* > 0.05), involving teeth # 43, 31, and 33 was observed.

**Discussion:**

The study's findings underscore a significant correlation between periodontitis and reduced pulp volume, suggesting that periodontal inflammation may influence pupal remodeling.

## 1 Introduction

Periodontal disease is a bacterially induced chronic inflammatory disease that impacts the periodontium, the supporting tissues of the teeth. It can lead to irreversible damage to the tooth's anchorage mechanisms and support system in the alveolar socket, potentially compromising the dental pulp (1). If untreated, the condition is likely worsen progressively and is currently considered the leading cause of tooth loss (2).

Endodontic lesions are infections within the tooth's pulp and root canal system (3), often arising from bacterial invasion due to dental caries, trauma, or other factors (4). When endodontic and periodontal lesions are present on the same tooth, it underscores a significant relationship between these conditions. The American Academy of Periodontology classifies these combined lesions as follows (5): (1) Retrograde periodontal disease, with subcategories including (a) Primary endodontic lesion with drainage through the periodontal ligament, and (b) Primary endodontic lesion with secondary periodontal involvement; (2) Primary periodontal lesion; (3) Primary periodontal lesion with secondary endodontic involvement; (4) Combined endodontic-periodontal lesion; and (5) Iatrogenic periodontal lesion.

Endodontic and periodontal lesions can be linked to the pulp tissue and periodontium (6). These structures may interact pathologically through fractures, cracks or physiologically through apical, lateral, and accessory canals, tubules. This connection facilitates the mutual transmission of inflammatory and degenerative processes, explaining the pathological relationship between pulp and periodontal tissues. Additionally, it sustains vascular, neurological, and sensory connections between these structures (7, 8).

Furthermore, in case of decay and cavities, a defense mechanism may activate on the surface of the pulp (9). For protection, the pulp generates secondary or tertiary dentin in the affected area, narrowing the pulp chamber (10). Additionally, pulp irritation can lead to the formation of pulpal calcifications and stones (11). Bacteria may originate in the pulp and spread through these anatomical pathways, eventually leading to a periodontal lesion (12, 13).

The impact of periodontitis on the dental pulp health remains unclear, despite extensive research over the past few decades. Numerous histological, radiographic, and clinical investigations have examined how periodontal disease might induce histological changes in the pulp. Findings, however, have been inconsistent; while some studies report normal pulp tissue, others reveal severe necrosis, chronic inflammation and degenerative manifestations including pulp calcifications (14). These calcifications, often manifesting as nodular deposits in the pulp cavity or diffuse calcifications within the root canals, are typical degenerative responses (14). Pulp stones are also considered a form of degeneration, defined as calcified masses within the pulps of healthy, diseased, and un-erupted teeth, where they may exist freely in the pulp or be attached to or embedded in the dentin (15).

Despite promising findings, two-dimensional (2D) radiographic imaging of the dental pulp has criticism for its limitations. Since it combines only the horizontal and parallel portions of the tooth, 2D imaging fails to capture the full extent of morphologic changes within the pulpal cavity that would be visible in three-dimensional (3D) (16). Advance imaging technologies, such as micro-computed tomography (CT) and cone-beam computed tomography (CBCT), offer non-invasive 3D visualization of dental structures (17). Unlike traditional 2D imaging, CT and CBCT use minimal radiation, making them safer for patients (18). CBCT, in particular, has gained popularity due to its reduced radiation exposure, capability for 3D image overlays, and enhanced imaging of critical areas, like the pulp and periapical regions (19). Furthermore, CBCT images can be transferred to a computer programs, allowing dentists to assess and monitor post-treatment changes, thus improving treatment planning and evaluation (19). Overall, CT and CBCT provide a safer and more effective way to obtain precise dental images (16). These advancements have transformed dental diagnostics and management, enabling more accurate assessments of various dental conditions, such as pulpal changes associated with periodontitis.

The rationale for assessing pulpal changes in periodontitis patients using CBCT through volumetric analysis lies in the intricate relationship between periodontal disease and pulpal health. As the reciprocal impact of periodontal disease on pulp tissue remains a significant area of uncertainty in dental research. While the effect of pulp on periodontal tissue has been extensively studied, the specific influence of periodontal disease on pulp volume and integrity remains unclear. This gap in knowledge hinders our ability to fully understand the interplay between periodontal health and pulp health. Consequently, there is a need to investigate whether periodontal disease significantly affects pulp volume. Therefore, traditional imagining modalities are used for the diagnosis however, traditional imaging modalities often fall short of providing comprehensive insights into the 3D architecture and volumetric changes of the pulp chamber and surrounding structures. CBCT offers enhanced visualization capabilities, allowing for accurate measurement of pulp volume and identification of pathological changes that may not be apparent on conventional radiographs. By integrating volumetric analysis, this study aims to bridge this knowledge gap by: (1) identifying the potential impact of periodontal disease on pulp volume using advanced imaging techniques, and (2) investigating the relationship between periodontal disease and pulpal changes. Furthermore, these can lead to improved diagnostic capabilities, tailored treatment approaches, and ultimately enhance patient outcomes in dental care.

## 2 Material and methods

### 2.1 Study design and participant selection

This study adopted a cross-sectional design and secured the approval from Institutional Review Board (IRB) on 28 January, 2024 (approval no. 4592420), focusing on patients who underwent CBCT for pre-operative assessment of implants, rather than for endodontic purposes. Participants who met the following inclusion criteria were considered eligible for the study: (1) the presence of periodontitis; (2) absence of cavity, abrasion, erosion, attrition, or restoration in the teeth; (3) teeth with bone loss not exceeding two-thirds of the length of the affected dental root; and (4) inclusion of anterior teeth in the evaluation.

Meanwhile, the exclusion criteria for participant selection included: (1) presence of endodontic lesions; (2) teeth with bone loss exceeding two-thirds of the root length;(3) exclusion of posterior teeth from the study; and (4) patients diagnosed with hypertension.

These criteria aimed to ensure a homogeneous and focused participant group for the study, facilitating accurate assessment of the impact of periodontal disease on pulp volume in the context of implant preoperative evaluations. For the numbering of teeth, the Zsigmondy-Palmer system was used.

### 2.2 Sample size determination

The following simple formula was used for calculating the adequate sample size (20);

$$n=Z^{2}(1-P)/d^{2}$$

Where *n* indicates sample size, *Z* is the statistic corresponding to the level of confidence, *p* is expected prevalence, and *d* is precision (corresponding to effect size). After calculations, this study employed 148 participants. Additionally, demographic data was also collected, including the participants' gender and age range (20–70 years). These demographic factors were integrated into the study's analysis to provide a comprehensive understanding of the study population.

### 2.3 Pulp-volume analysis

An in-depth analysis was performed on anterior teeth from canine to canine in maxilla and mandible (13, 12, 11, 21, 22, 23, 41, 42, 43, 31, 32, and 33) to assess their pulp volume and related parameters using CBCT imaging and Amira software and measurements were derived using bio-models generated from CBCT images.

### 2.4 Ethical considerations

This study was conducted in accordance with ethical guidelines and principles outlined in the Declaration of Helsinki, as revised by the General Assembly in October 2013. The research protocol was reviewed and approved by the IRB of King Abdulaziz University Faculty of Dentistry, ethical approval No. (4592420). Informed consent was obtained from all participants prior to their participation in the study, and The confidentiality of personal data was strictly maintained throughout the research process.

### 2.5 Statistical analysis

**Figure 1:**
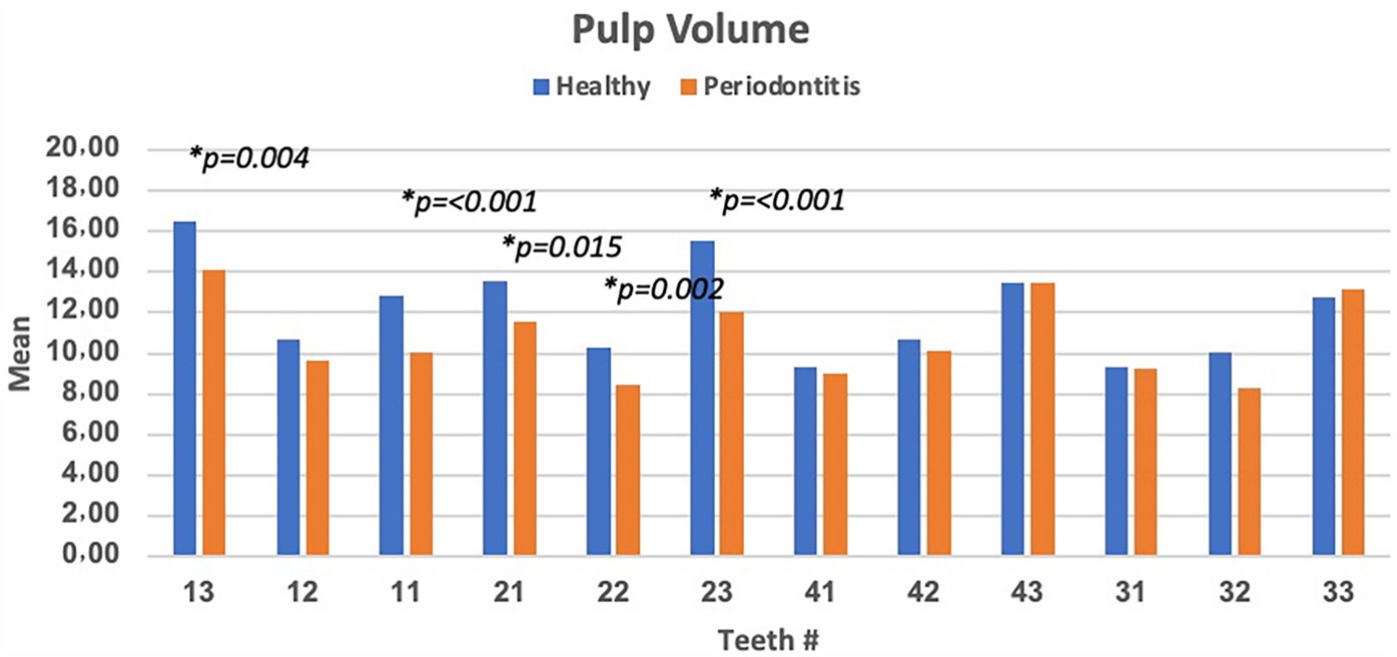
Represent the pulp volume determination. This figure illustrated the quantification process involved reconstructed volumetric data, which provided insights into the structural characteristics of the pulp as highlighted in the red colour in the right panel of the figure.

Descriptive statistic was performed to define the characteristics of the study variables in the form of frequencies and percentages for the categorical and nominal variables while continuous variables were presented by mean and standard deviations (SD). To establish a relationship between periodontal diagnosis and the pulp volume of each tooth, an independent *t*-test was used for comparing two group means, and for more than two groups a One-way ANOVA was used. These tests were done with the assumption of normal distribution. Otherwise, Welch's *t*-test for two group means was used as an alternative test. Meanwhile, a *p*-value was set at <0.05 and all the data was analyzed using IBM SPSS version 27 (IBM Corp., Armonk, N.Y., USA). Furthermore, utilizing a series of scripts within the Amira software, bio-models derived from the CBCT images were generated. These scripts facilitated the importation of Digital Imaging and Communications in Medicine (DICOM)-formatted images into the software, allowed for masking with appropriate values, and enabled the subsequent creation of 3D images through 3D analysis. Initially, the tooth pulp tissue boundaries were defined using bone Hounsfield Unit (HU) values (−1,000 to 4,000) to generate 3D images from the DICOM data. Once the requisite HU values were established, the pulp tissues were isolated from surrounding tissues by drawing points through the pulp, which were then automatically processed by the software. Subsequently, the volume of the pulp tissues was quantified post-creation of the 3D images (Figure 1).

## 3 Results

### 3.1 Characteristics of the study participants

Table 1 summarizes the characteristics of the study patients (*N* = 148). The mean age of the patients was 39.51 ± 13.2, with the youngest being 20 years old and the oldest 70 years. The gender distribution was almost equal, with 77 (52%) male and 71 (4%) female patients with no history of hypertension. Among the 148 patients, nearly half (73; 49.3%) were diagnosed with periodontitis, and the other half (75; 50.7%) were considered healthy. The majority of the patients with periodontitis were classified as stage III (*n* = 44, 29.7%) and Grade C (31, 20.9%). The details of the patient diagnosis are shown in Table 1.

**Table 1:** Summary of general characteristics of patients (*N* = 148).

Demographics	N	Min	Max	Mean	SD
Age	148	20	70	39.51	13.2
		Frequency		Percentage	
Total		148		100.0	
HTN	No	148		100.0	
Gender	Male	77		52.0	
	Female	71		48.0	
Perio diagnosis	Healthy	75		50.7	
	Stage I grade B	3		2.0	
	Stage I grade C	1		0.7	
	Stage II grade B	6		4.1	
	Stage II grade C	7		4.7	
	Stage III grade B	13		8.8	
	Stage III grade C	31		20.9	
	Stage IV grade C	12		8.1	
Perio diagnosis	Healthy	75		50.7	
	Stage I	4		2.7	
	Stage II	13		8.8	
	Stage III	44		29.7	
	Stage IV	12		8.1	
Perio diagnosis	Healthy	75		50.7	
	Periodontitis	73		49.3	

HTN, hypertension; N, total number; SD, standard deviation.

### 3.2 Pulp volume analysis

The results revealed that the mean low pulp volume was found with mandibular incisors (9.15 ± 3.3 mm^3^ for teeth #41 followed by teeth # 31 and 32 (9.28 ± 4.1 and 9.34 ± 3.6 mm^3^) and higher volume was with canine teeth (15.24 ± 4.2 mm^3^ for teeth # 13, followed by teeth # 23 (14.06 ± 3.9 mm^3^), and teeth # 43 (13.46 ± 3.1 mm^3^) as indicated in Table 2.

**Table 2:** Summary of pulp volume of the study patients.

Pulp volume	N	Min	Max	Mean	SD
Teeth #13	101	3.203	30.159	15.24	4.2
Teeth #12	98	5.184	26.816	10.21	3.7
Teeth #11	89	5.238	17.760	11.35	3.3
Teeth #21	94	3.203	23.220	12.63	3.9
Teeth #22	91	3.203	16.554	9.41	2.9
Teeth #23	93	3.203	23.625	14.06	3.9
Teeth #41	88	3.127	17.088	9.15	3.3
Teeth #42	79	3.203	19.584	10.44	3.4
Teeth #43	81	4.160	19.584	13.46	3.1
Teeth #31	82	3.942	26.816	9.28	4.1
Teeth #32	76	4.160	17.088	9.34	3.6
Teeth #33	92	4.224	26.816	12.94	4.0

SD, standard deviation.

### 3.3 Comparison of pulp volume between stages classification

Pulp volume involving teeth # 13 (*p* = 0.001), teeth # 22 (*p* = 0.026), teeth # 43 *(p* = 0.013) and teeth #32 (*p* = 0.003) were found to be significantly different across various periodontal stages (Table 3). While, pulp volume involving teeth #11 and 23 were found to be highly significantly (*p* < 0.001) different across different stage classifications of periodontitis. These results underline substantial fluctuations in pulp volume across different stages of periodontal disease for these specific teeth. However, no-significant difference (*p* > 0.05) in the pulp volume means across various stages involving teeth #12, 21, 41, 42, 31, and 33 was also observed (Table 3).

**Table 3:** Pulp volume of patients across stages of periodontitis.

Pulp volume	Total	Healthy	Stage I	Stage II	Stage III	Stage IV	Power	*p*-value
Teeth #13	101	16.50 ± 3.5	9.85 ± 5.8	17.61 ± 4.0	13.85 ± 4.2	13.20 ± 5.1	0.950	0.001*
Teeth #12	98	10.72 ± 2.9	6.34 ± 0.0	9.87 ± 3.7	10.07 ± 4.7	9.31 ± 4.3	0.366	0.313
Teeth #11	89	12.79 ± 2.8	10.66 ± 0.0	8.38 ± 3.0	9.87 ± 3.2	11.41 ± 3.2	0.977	<0.001*
Teeth #21	94	13.51 ± 3.5	0.00 ± 0.0	10.85 ± 2.9	11.80 ± 4.5	11.10 ± 3.0	0.532	0.099
Teeth #22	91	10.31 ± 2.5	0.00 ± 0.0	8.29 ± 4.4	8.51 ± 2.9	8.43 ± 3.0	0.728	0.026*
Teeth #23	93	15.51 ± 3.0	12.96 ± 0.0	11.13 ± 5.2	12.22 ± 4.2	13.28 ± 3.1	0.972	<0.001*
Teeth #41	88	9.30 ± 2.3	7.96 ± 0.0	9.52 ± 4.6	9.15 ± 4.7	8.26 ± 3.7	0.097	0.924
Teeth #42	79	10.71 ± 2.6	14.07 ± 0.0	11.72 ± 5.1	9.61 ± 3.9	9.57 ± 4.6	0.306	0.406
Teeth #43	81	13.43 ± 1.5	0.00 ± 0.0	8.83 ± 5.1	14.03 ± 4.5	14.67 ± 4.2	0.800	0.013*
Teeth #31	82	9.34 ± 2.8	0.00 ± 0.0	11.25 ± 4.6	9.28 ± 6.1	7.50 ± 3.9	0.257	0.408
Teeth #32	76	10.02 ± 2.4	8.13 ± 0.0	15.63 ± 1.8	8.42 ± 4.5	6.18 ± 4.5	0.927	0.003*
Teeth #33	92	12.78 ± 2.8	15.94 ± 0.0	10.95 ± 2.4	12.84 ± 4.8	14.68 ± 7.2	0.258	0.504

* Significant using One-Way ANOVA Test at <0.05 level.

### 3.4 Comparison of pulp volume between healthy and diagnosed patients

The differences of pulp volume between healthy teeth and those affected by periodontitis were also analyzed and summarized in Table 4. Statistical analysis revealed a significant difference in pulp volume between healthy teeth with higher mean pulp volume (16.50 ± 3.5) and those diagnosed with periodontitis (14.10 ± 4.6) involving teeth # 13 (*p* = 0.004), teeth # 23 (15.51 ± 3.0 vs. 12.05 ± 4.2, *p* < 0.001), teeth # 21 (13.51 ± 3.5 vs. 11.55 ± 4.1, *p* = 0.015), and teeth # 22 (10.31 ± 2.5 vs. 8.46 ± 3.1, *p* = 0.002). Based on Welch's *t*-test result, a highly significant difference in pulp volume between healthy and teeth with periodontitis was found involving only at teeth # 23 (*p* < 0.001). Meanwhile, a non-significant difference (*p* > 0.05) in the least pulp volume was observed in teeth # 41 (9.30 ± 2.3 vs. 8.98 ± 4.3), followed by 31 (9.34 ± 2.8 vs. 9.21 ± 5.4). The highest non-significant difference in the pulp volume was observed in teeth # 43 (13.43 ± 1.5 vs. 13.50 ± 4.7), 33 (12.78 ± 2.8 vs. 13.15 ± 5.2) (Table 4).

**Table 4:** Pulp volume between healthy and teeth with periodontitis of patients.

Pulp volume	Total	Healthy	Periodontic	Power	*p*-value
Teeth #13	101	16.50 ± 3.5	14.10 ± 4.6	0.834	0.004*
Teeth #12	98	10.72 ± 2.9	9.68 ± 4.3	0.280	0.165
Teeth #11	89	12.79 ± 2.8	10.06 ± 3.1	0.990	<0.001*
Teeth #21	94	13.51 ± 3.5	11.55 ± 4.1	0.686	0.015*
Teeth #22	91	10.31 ± 2.5	8.46 ± 3.1	0.875	0.002*
Teeth #23	93	15.51 ± 3.0	12.05 ± 4.2	0.993	<0.001**
Teeth #41	88	9.30 ± 2.3	8.98 ± 4.3	0.071	0.673
Teeth #42	79	10.71 ± 2.6	10.13 ± 4.2	0.112	0.465
Teeth #43	81	13.43 ± 1.5	13.50 ± 4.7	0.051	0.935
Teeth #31	82	9.34 ± 2.8	9.21 ± 5.4	0.052	0.899
Teeth #32	76	10.02 ± 2.4	8.29 ± 4.8	0.486	0.074
Teeth #33	92	12.78 ± 2.8	13.15 ± 5.2	0.070	0.688

* Significant using Independent *t*-test at <0.05 level.

** Significant using Welch's *t*-test at <0.05 level.

**Figure 2:**
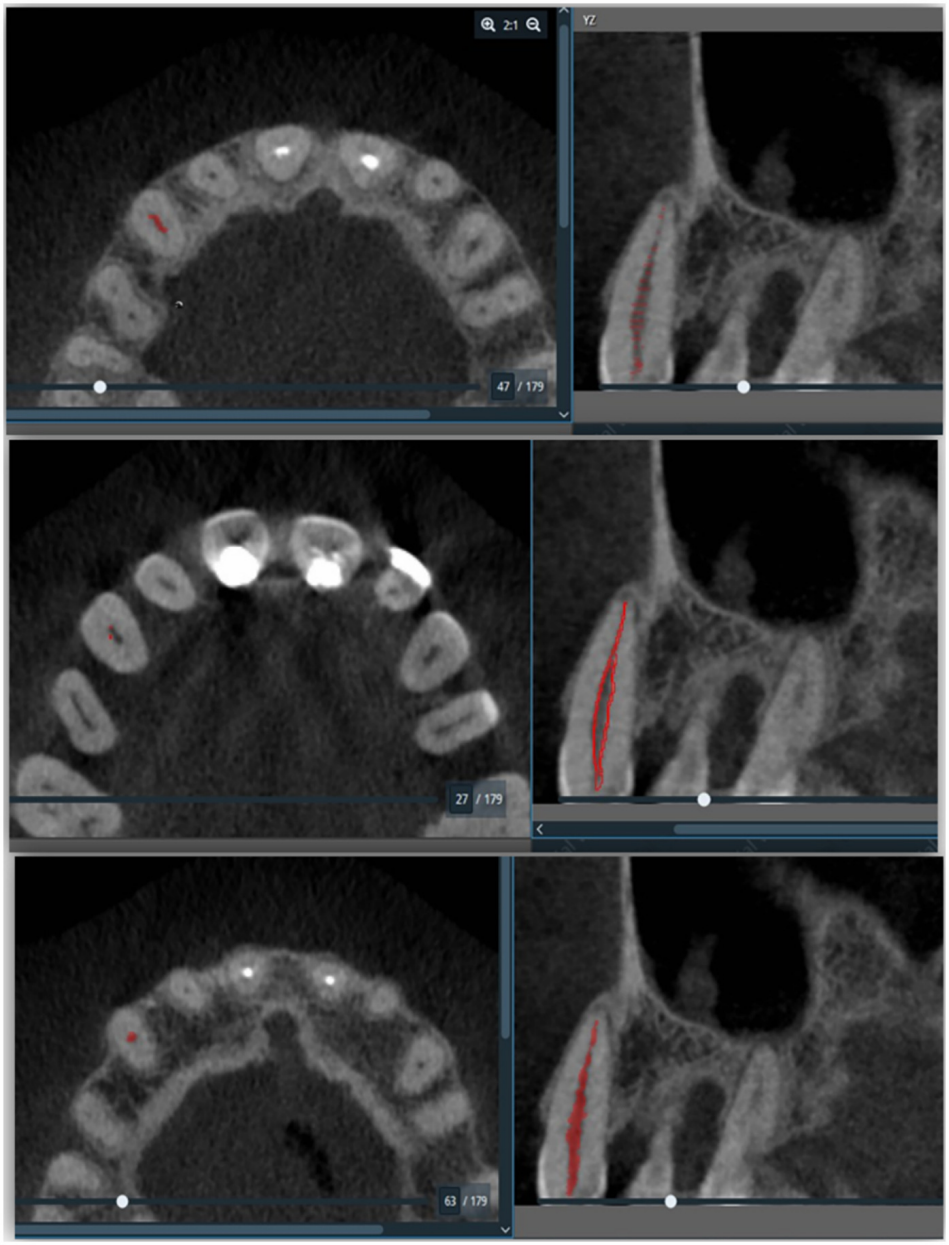
Pulp volume differences between healthy and teeth with periodontitis involving different teeth numbers and significant difference (*p* < 0.05) was observed in the teeth # 13, 11, 21, 22, and 23).

The mean pulp volume between healthy and teeth with periodontitis showed certain differences. The highest significant difference (*p* < 0.05) was observed in involving teeth # 23, 11, and 13. However, non-significant difference (*p* > 0.05) were observed involving teeth # 43, 31, and 33. Furthermore, higher mean of pulp volume was observed in healthy teeth than in periodontitis-diagnosed teeth except for teeth # 33 and 43 (Figure 2).

## 4 Discussion

There is a lot of controversy surrounding the potential link between pulp and periodontal tissues. The same mechanism that causes inflammation in other tissues also occurs in dental pulp. This association is attributed to the blood flow disruption and essential nutrient supply to the pulp due to periodontal disorders, leading to a reduction in cellular components and an increase in calcifications (21). Furthermore, the pulp can exhibit a variety of degenerative changes in response to stimulation, including calcifications and fibrosis (22). Moreover, the differences in pulpal tissue changes between extracted teeth with advanced periodontitis and those with a healthy periodontium were investigated. Meanwhile severe periodontitis does not directly lead to pulpal necrosis or significant changes in pulp vitality and calcifications, it does contribute to pulp fibrosis, apical inflammation, and internal and external resorption (23). Furthermore, the literature suggests that teeth with CAL values up to 8 mm and PD depths of 5 mm, which do not extend to the apex, may exhibit a negative response to pulpal stimuli. Additionally, periodontal changes do not need to extend to the vicinity of the apical foramen to establish a connection between periodontal and pulpal tissues (24).

In the present study, a significant difference (*p* < 0.05) and higher mean in pulp volume between healthy teeth and those diagnosed with periodontitis for teeth #13, followed by 23, 22, and 11 was observed. A higher means of pulp volume was also observed in healthy teeth than in periodontitis-diagnosed teeth except for teeth # 33 and 43. This finding could indicate a possible response of the pulp tissue to periodontal disease, potentially involving compensatory mechanisms or variations in inflammatory responses. Meanwhile, there are other studies align with our findings as in a histological study performed by Gautam et al. (25) using extracted teeth from patients with severe periodontitis, it was discovered that degenerative changes such as inflammation, fibrosis, edema, calcification, and necrosis were present to varying degrees in the presence of moderate to severe chronic periodontitis. This emphasizes the relationship between periodontal disease and pulpal health. Similarly, another study also highlighted a substantial impact of dental pulp tissue on both pulp volume and surface areas, leading to a notable 20% reduction in pulp volume (16). There is another compelling evidence indicating that teeth affected by periodontal disease can lead to inflammation and degeneration of the dental pulp. Studies found a correlation between clinical attachment loss, depth of periodontal probing, and gingival recession with adverse pulpal reactions, which are notably influenced by the advancement of periodontitis (24). Moreover, the state of the pulp is associated to the severity of ongoing periodontal disease (26). The findings of the present study reinforce this notion, suggesting that periodontal defects have an impact on both the volume and surface area of the pulp. These findings mirrored the significant differences observed in pulp volume between healthy teeth and those affected by periodontitis, which aligns with the observations for teeth #12, 41, 42, 43, 31, 32 and 33. Furthermore, these findings underscore a consistent pattern of reduced pulp volume in teeth impacted by periodontitis compared to healthy teeth. This agreement in outcomes strengthens the understanding of how periodontitis influences alterations in pulp volume, highlighting a direct correlation between the severity of periodontal disease and changes in pulp health.

In the present study, the significant and higher and lower mean differences (*p* < 0.05) observed in the pulp volume of teeth # 13 and 32, respectively across different stages of periodontal disease may be attributed to several factors, as highlighted by the findings from stages II to IV. Tooth #13 exhibited significant differences with higher values of 17.61 ± 4.0 for stage II, and decreasing with 13.85 ± 4.2 for stage III, and 13.20 ± 5.1 for stage IV. This decreasing trend in pulp volume from stage II to stage IV may reflect progressive inflammatory changes and tissue damage, leading to a reduction in pulp volume. Additionally, tooth # 32 displayed significant differences with higher values of 15.63 ± 1.8 for stage II, and decreasing with 8.42 ± 4.5 for stage III, and 6.18 ± 4.5 for stage IV (*p* = 0.003). This notable decrease in pulp volume from stage II to stage IV for teeth # 13 and 32 indicates significant tissue alterations associated with advanced periodontal disease stages. These fluctuations in pulp volume across different stages of periodontal disease for these specific teeth underscore the impact of periodontitis progression on pulp health, highlighting the relationship between periodontal health and pulp integrity. Furthermore, the chronic inflammatory environment in periodontitis can induce dystrophic calcifications within the pulp chamber, contributing to pulp calcifications and ultimately impacting the vitality and function of the pulp tissue (14). Our findings particularly for teeth # 13 and 32, mirror the observations of Zuza et al. (21) and Terlemez et al. (16) as they demonstrated a significant decrease in pulp chamber volume with increasing severity of periodontitis, indicating a direct impact of inflammatory processes from periodontitis on pulp health. Similarly, another study also revealed significant (*p* < 0.05) decrease in the pulp space volume (27). Conversely, a radiological analysis evaluated 332 teeth from records of 79 patients with periodontitis. Using periapical preoperative radiographs, they compared 81 intact dental crowns with attachment loss to their contralateral intact dental crowns without any attachment loss. The study found no correlation between pulp calcifications and the occurrence of attachment loss (15). Similarly, according to Bains et al. (28), cementum acts as a barrier preventing septic substances from moving from the periodontal pocket to the pulp. Despite the progressive destruction of the periodontal attachment system, bacteria in dental plaque and associated substances can escape through the coronal part of the pocket, while the cementum barrier prevents their direct passage into the endodontium (28). These findings align with the results reported by Alattas (29) which similarly observed trends in pulp volume alterations related to the progression of periodontal disease. Therefore, this can explain the result of the remaining teeth such as teeth # 33 and 43 which showed non-significant difference between healthy and periodontitis patients. These findings underscore the importance of assessing pulpal changes in periodontitis patients, with CBCT-based volumetric analysis proving to be a valuable tool in understanding the complex interplay between periodontal disease and pulpal health.

To determine whether there are any variations in the link between tooth surface area and pulp volume, future research should investigate this relationship. Researchers can obtain a full understanding of tooth-specific changes and their effect on pulpal health in patients with periodontitis by combining surface area analysis with pulp volume measurements. This method may provide subtle insights into how variations in pulp volume may be influenced by changes in tooth morphology, which would ultimately improve our capacity to customize treatment plans for specific patients. Investigating this association may provide important information regarding dental diagnosis and treatment planning, which could result in more accurate and successful management of periodontal disease and its related pulpal complications.

One limitation of the study may be the challenge encountered in obtaining samples of healthy teeth that had not undergone restoration or filling procedures. This difficulty arises because such teeth are less common in clinical practice, especially among patients who had sought dental care previously. Consequently, the study's ability to include a sufficient number of patients, and untreated teeth for comparison purposes may be limited. This limitation could potentially impact the generalizability of the findings, as the study sample may not fully represent the broader population of teeth unaffected by restoration or filling interventions.

## 5 Conclusion

This study enhances our understanding of the relationship between pulp and periodontitis. Established methods such as radiography, histology, or their combination, alongside clinical assessments, remain crucial in effectively determining this link despite ongoing efforts to develop advanced diagnostic technologies. The CBCT enables a comprehensive assessment of lesion extent and the development of tailored treatment plans addressing both endodontic and periodontal aspects. Throughout these processes, prioritizing patient comfort and safety remains imperative, emphasizing the importance of thorough examination and analysis in dental practice.

## Data Availability

The raw data supporting the conclusions of this article will be made available by the authors, without undue reservation.
